# Yeast Ecology in White Brined Cheeses: Correlations with Physicochemical Parameters in Artisanal and Industrial Products

**DOI:** 10.3390/microorganisms13091965

**Published:** 2025-08-22

**Authors:** Neli Ermenlieva, Sylvia Stamova, Nadezhda Ivanova, Petya Atanasova, Velichka Marinova, Sevginar Ibryamova, Ivan Ivanov, Emilia Georgieva

**Affiliations:** 1 Department of Microbiology and Virusology, Faculty of Medicine, Medical University, 9000 Varna, Bulgaria; 2 Department of Pharmaceutical Chemistry, Faculty of Pharmacy, Medical University, 9000 Varna, Bulgaria; silvia.stamova@mu-varna.bg; 3 Department of Pharmaceutical Technologies, Faculty of Pharmacy, Medical University, 9000 Varna, Bulgaria; nadejda.ivanova@mu-varna.bg; 4 Professor Dr. Asen Zlatarov Vocational High School of Tourism, 9000 Varna, Bulgaria; petya.atanasova@pgtvarna.com; 5 Department of Commodity Science, Faculty of Economics, University of Economics, 9000 Varna, Bulgaria; velichka.peewa@abv.bg; 6 Faculty of Natural Sciences, Department of Biology, Shumen University, 9700 Shumen, Bulgaria; s.ibryamova@shu.bg; 7 Regional Directorate for Food Safety, Head of Laboratory Activities Department, 9000 Varna, Bulgaria; ivan_st67@mail.bg; 8 Training Sector Medical Laboratory Technician, Medical College–Varna, Medical University, 9000 Varna, Bulgaria; emiliya.georgieva@mu-varna.bg

**Keywords:** white-brined cheese, wild yeast microbiota, *D. hansenii*, *T. delbrueckii*, *S. cerevisiae*

## Abstract

Yeasts are essential contributors to the ripening and flavor development of white brined cheeses. This study aimed to investigate and compare the microbial load and yeast species composition in artisanal and industrial white brined cheeses. The influence of key physicochemical parameters (salt content, acidity, fat content, moisture, and ripening stage) on yeast count and species composition was analyzed. A total of 100 white brined cheese samples produced in Bulgaria were analyzed. Yeast species were identified using MALDI–TOF MS, and physicochemical properties were assessed according to ISO standards. The predominant yeast species identified were *Torulaspora delbrueckii*, *Debaryomyces hansenii*, *Saccharomyces cerevisiae*, and *Candida sphaerica*. *D. hansenii* was the dominant species in industrial samples, while *S. cerevisiae* was more frequently isolated from artisanal cheeses. Statistical analyses showed that the physicochemical parameters most influencing yeast species composition were salt content and acidity. A statistically significant correlation between yeast count and salt content was observed only in industrial cheeses, with *D. hansenii* showing greater salt tolerance. Yeast counts were higher in cheeses with higher salt content, particularly in industrial samples. This study highlights the distinct influence of production methods and physicochemical parameters on the yeast ecology of white brined cheeses.

## 1. Introduction

Cheese is a major dairy product in the European Union, where over 39% of whole milk is used for its production [1]. With a global output of about 20 million tons, the EU remains the top exporter [2]. In Bulgaria, cheese and curd exports reached 21.8 thousand tons in 2023, making up over 53% of total dairy exports [3].

Industrially produced cheeses are manufactured in large quantities, following standardized processes, including pasteurization, that ensure consistent quality. These cheeses provide all the essential nutrients found in cheese while also offering convenience at an affordable price, making them accessible to a broad consumer base [4]. In contrast, various types of artisanal cheese are produced from raw milk, with the microbiota of the raw milk playing a crucial role in shaping the microbial composition of the final cheese [5]. Artisanal cheeses are produced locally, often following traditional regional recipes and using conventional cheesemaking techniques, typically in small-scale production. The milk used for their production is sourced from a single or a limited number of farms, ensuring a strong connection to their region of origin. These cheeses are deeply influenced by the local terroir, encompassing factors such as climate, landscape, rural development, and the knowledge and expertise of local producers [6,7]. The traditionally produced artisanal cheeses are characterized by increased microbial diversity associated with superior flavor, aroma, and texture, while the standardized manufacturing of industrial cheeses leads to a less complex microbiota in an attempt to improve product safety at the expense of sensorial quality [5].

The complex microbial ecosystem of cheese comprises both starter cultures [8,9], and adventitious bacteria, yeasts, and molds that unintentionally enter the product [10,11]. The primary research focus is on the contribution of lactic acid bacteria to cheese ripening and quality. The metabolic influence of yeasts on cheese characteristics was long underestimated. However, over the past three decades, knowledge about the role of yeasts in cheese ripening has expanded significantly [11,12,13]. The ability of yeasts to utilize components of dairy raw materials enables their application in the dairy industry. Yeasts are facultative anaerobes; they develop and multiply in the presence of oxygen and actively carry out fermentation in its absence, accumulating ethyl alcohol and releasing CO_2_. The deacidification of cheese by yeasts begins at the surface of the product, creating a pH gradient from the exterior toward the center and causing lactate diffusion outward. Once lactate is depleted, yeasts catabolize amino acids, producing NH_3_, which diffuses inward, further increasing alkalinity. This pH elevation is a crucial prerequisite for cheese ripening. Consequently, the role of yeasts in the maturation process is now widely recognized [13].

Yeasts are not nutritionally demanding microorganisms and, compared to bacteria, grow more slowly and usually do not compete directly with them. They thrive in acidic environments where bacterial growth is either inhibited or significantly reduced. Consequently, the low pH of freshly made cheese acts as a partially selective factor for yeast growth, in contrast to the majority of bacterial species [4,14]. The presence of these microorganisms in cheese is beneficial due to their ability to proliferate and tolerate low pH, high salt concentrations, reduced water activity, and low temperatures [13,15,16]. These microorganisms accelerate the ripening process by increasing the pH on the cheese surface through the metabolism of lactate into CO_2_ and H_2_O. Many yeast species exhibit proteolytic activity, releasing free amino acids [17,18]. They produce desirable aromatic compounds such as alcohol, methyl ketones, and lactones, which can enhance the flavor profile of cheeses [19]. They also contribute to extending the shelf life of cheese due to their ability to inhibit the growth of undesirable microorganisms [20].

The diversity and abundance of specific microbial species present in cheese depend on the microbial quality of the milk, as well as its processing, including thermal treatment. Other factors influencing the yeast count and microbial diversity in cheese include the conditions of curd production, ambient humidity and temperature during ripening, the amount of salt used in the cheese and/or brine, the exposure of the cheese to exogenous microorganisms throughout these stages and post-production [8,21]. The diversity and concentration of yeast in cheese microbiota depend on a complex interplay of intrinsic factors (such as raw material quality and milk microbiota), extrinsic factors (processing, preservation conditions, and environmental influences), and biological factors, including interactions between indigenous and introduced microorganisms. These combined influences lead to considerable variability in the genera and species present in the final product.

The factors influencing yeast communities in cheese are not identical in industrial and farmhouse production. Industrial production of white brined cheese typically uses pasteurized milk, which eliminates most natural yeasts and microorganisms. Ripening occurs under controlled conditions (12–14 °C), resulting in a more uniform flavor profile dominated by ketones, aldehydes, and esters. The role of yeasts in industrial production is relatively limited, contributing primarily to texture and mild flavor. In contrast, traditional farmhouse production relies on raw milk, which preserves the natural microbial flora, including a diverse range of yeasts. Fermentation is often driven by the natural microbiota or supplemented with natural starter cultures. Aging takes place under natural conditions (8–14 °C), leading to a more complex flavor profile with fruity, buttery, and fusel notes. Yeasts play an active role in flavor and aroma development in farmhouse cheese, contributing to its distinctive sensory characteristics. Processing conditions not only determine the presence or absence of native microorganisms but also modulate the interactions between yeasts and autochthonous bacteria, affecting microbial succession, metabolic cooperation, and overall ripening dynamics [8,13,21]. In countries such as Greece, Turkey, and France, certain yeast species, including *Debaryomyces hansenii* and *Kluyveromyces lactis*, are intentionally added to cheese recipes to enhance the flavor, aroma, and texture of the cheese [12,22]. However, yeasts are not part of the traditional recipe for white brined cheese in many countries. The rennet used in white brined cheese production contains the enzymes essential for milk coagulation but does not include yeast.

The total yeast count in raw milk typically ranges from 10 to 10^3^ CFU/mL [18]. According to [13] *Kluyveromyces* spp., *G. candidum*, *Yarrowia lipolytica*, *D. hansenii*, and *Pichia* spp. are among the most commonly found yeast species in raw milk. These same species have also been isolated from autochthonous whey and milk starter cultures used in cheese production. Other authors additionally report the presence of *Geotrichum candidum*, *Torulaspora delbrueckii,* and the genera *Candida*, *Cryptococcus*, *Trichosporon*, and *Rhodotorula* spp. [23,24,25].

The most frequently isolated yeast species in white brined cheese are *T. delbrueckii* (anamorph *Candida colliculosa*), *Saccharomyces cerevisiae*, *D. hansenii* (anamorph *Candida famata*), *K. lactis* (anamorph *Candida sphaerica*), *Candida zeylanoides*, and *Pichia fermentans* (anamorph *Candida lambica*). These species have been consistently reported across different investigations, suggesting that they play a key role in shaping the microbial community of white brined cheese [26,27,28,29,30,31]. Less frequently detected species include *Candida intermedia*, *Geotrichum candidum*, *Kluyveromyces marxianus*, *Pichia guilliermondii*, *Pichia membranifaciens*, and *Rhodotorula* spp., which have been identified sporadically in certain samples [30,32,33].

Yeasts and molds are generally not considered common causes of foodborne contamination; however, certain mold species may produce mycotoxins, and some yeasts can cause undesirable off-flavors or odors in cheese or produce metabolic compounds that reduce cheese quality and may pose a potential health risk [34]. Some of these yeasts have been reported as opportunistic pathogens, particularly in immunocompromised patients. This is especially relevant for *Candida* species. Medically significant yeast species found in various cheeses primarily include *Candida albicans*, *Candida tropicalis*, *Candida krusei*, and *Candida glabrata* [8]. Several opportunistic yeasts, such as *Candida catenulata*, *Candida parapsilosis*, *C. krusei*, *Candida tropicalis*, and *Kodamaea ohmeri*, have also been repeatedly detected in cheese [8]. While the presence of these fungi in dairy products is not uncommon, invasive fungal infections resulting from their consumption are extremely rare and far less frequently reported in the literature than foodborne mold-related illnesses. Notably, all documented cases have been associated with dairy products [13].

The identification of yeasts in milk and dairy products has been well documented by [4] Traditionally, yeast identification has been carried out based on differences in colony morphology, microscopy, and phenotypic characteristics, such as growth requirements and carbohydrate metabolism [22,35]. However, these methods are complex and can produce ambiguous results. Over the past two decades, yeast identification has significantly advanced with the application of molecular genetic techniques [36,37,38]. Denaturing high-performance liquid chromatography (DHPLC) has also been employed for the identification of yeasts in cheese [39,40]. More recently, advanced techniques such as matrix-assisted laser desorption/ionization–time of flight mass spectrometry (MALDI–TOF MS) and Fourier transform infrared spectroscopy (FTIR) have been applied for the identification of dairy-associated yeasts [9,22,41]. While MALDI–TOF MS provides rapid and high-throughput identification, its accuracy may be improved by complementary biochemical and molecular genetic assays, particularly when distinguishing closely related or atypical strains [22]. MALDI–TOF MS generates protein-based spectral profiles, or “fingerprints,” obtained through the desorption of specific peptide/protein biomarkers released from the cell surface after acid treatment. In contrast, FTIR relies on the detection of functional biochemical groups directly from intact cells, producing metabolic spectral fingerprints unique to the yeast species [4,22].

These modern identification systems provide numerous new opportunities for more in-depth studies of yeast consortia in both industrial and farmhouse products obtained through traditional technologies across different geographical regions. Nevertheless, we found no studies investigating the composition and count of yeasts in these categories of white brined cheeses. Therefore, further research is needed to enhance the understanding of the yeast composition in both artisanal and industrially produced cheeses.

To the best of our knowledge, this is the first study that systematically compares the yeast microbiota of artisanal and industrial white brined cheeses produced in the same geographical region but under different technological conditions. The novelty of the research lies in the combined analysis of microbial counts, yeast species identification, and physicochemical parameters, which provides a comprehensive understanding of how production method influences yeast diversity and dynamics in white brined cheeses.

The aim of the study was to investigate and compare the microbial load and yeast species composition in artisanal and industrial white brined cheeses. Additionally, the study sought to differentiate the physicochemical characteristics of the cheese that influence the composition of yeasts and yeast counts in the two examined categories of white brined cheese.

## 2. Materials and Methods

### 2.1. Materials

In this study, the following chemicals and microbiological media were used. All chemicals were of analytical grade (a.r.), and microbiological media were of laboratory quality suitable for microbiological analysis. The chemicals used included silver nitrate (≥99.99% AgNO_3_), sulfuric acid (95–97%), sodium carbonate (anhydrous, ≥99.8% Na_2_CO_3_), and potassium chromate (≥99.5% K_2_CrO_4_), all sourced from ChemLab. Sodium hydroxide (≤97%) was purchased from Fisher Chemical, while thymolphthalein (ACS reagent) was supplied by Thermo Scientific (Waltham, MA, USA). A 1% solution of phenolphthalein in ethanol was obtained from Himtex.

For microbiological analysis, HiMedia(Maharashtra, India) provided the Maximum Recovery Diluent, DRBC agar (Dichloran Rose-Bengal Chloramphenicol Agar), and Blood agar, all of which were used in the preparation of microbial cultures.

GenAI was used to improve the English language for writing the article.

### 2.2. Cheese Samples

Bulgarian white brined cheese belongs to the category of semi-hard rennet cheeses. It is produced through the coagulation of milk casein using a rennet enzyme (cheese starter), followed by a maturation period and storage in brine.

A selection of 100 white brined cow’s milk cheeses produced in Bulgaria from Bulgarian cow’s milk was organized. A total of 50 samples were classified as artisanal cheeses, of which 29 were purchased from farms registered under Ordinance 26 [42], which regulates farmhouse cheese production. The remaining 21 samples were obtained from private dairies and farmers’ markets across different geographical regions of Bulgaria.

The other 50 cheeses were categorized as industrial cheeses and were purchased from supermarkets within the commercial retail network. None of the industrial cheeses were labeled by the manufacturer as artisanal or organic products, nor were they placed in the farmhouse or organic product section within the retail establishments.

All cheese samples analyzed in the study were purchased in vacuum packaging, had undergone a ripening period of at least 45 days, and were divided into two categories for the purposes of the study:

Artisanal cheeses—produced by manufacturers registered under Ordinance 26 or explicitly categorized as such by the producer.

Industrial cheeses—widely available in the commercial retail network and without any labeling indicating farmhouse or organic status.

After collection, the samples were transported to the laboratory and analyzed within 24 h.

### 2.3. Physicochemical Analysis

Titratable acidity was originally measured in degrees Thörner (°T) following the national standard method [43]. For clarity and broader comparability, the values were converted to grams per liter (g/L) of lactic acid using the following conversion: 1 °T = 0.09 g/L lactic acid. The degree of maturity (°Sh) was evaluated by the Shilovich method [44], which involves titration with 0.1 N NaOH using thymolphthalein and phenolphthalein as indicators. Dry matter content (%) was determined gravimetrically by drying the sample at 102 °C until a constant weight was reached, in accordance with ISO 5534 [45]. The salt content (%) was analyzed using the Mohr method, involving titration with 0.1 N AgNO_3_ in the presence of potassium chromate, as described in ISO 5943 [46]. The fat content (%) was measured using the Van Gulik method, where samples were treated with H_2_SO_4_ and isoamyl alcohol, centrifuged, and the fat percentage was read directly from the butyrometer scale, following ISO 3433 [47]. These methods ensure accurate and standardized assessment of the physicochemical properties of the cheese samples. All physicochemical analyses were performed in triplicate for each cheese sample to ensure accuracy and reproducibility of results.

### 2.4. Detection and Count of Yeasts

Each cheese sample was unpacked under aseptic conditions. For the detection and enumeration of the total yeast count, 10 g of the cheese sample were taken from both the interior and the rind (surface). Each 10 g sample was aseptically transferred using tweezers and mixed with 90 mL of Maximum Recovery Diluent (dilution 10^−1^). The suspension was homogenized for 1 min using a Stomacher Lab Blender 400 (Seward Laboratory Systems, Davie, FL, USA), and a series of tenfold dilutions was prepared up to 10^−7^. For each dilution, three replicate plates were inoculated and incubated to ensure accuracy and reproducibility.

From each dilution, 1 mL was plated onto the surface of DRBC agar (containing 0.5% yeast extract, 2% glucose, 1% agar, and 0.1% chloramphenicol) in 120 mm diameter Petri dishes. The samples were evenly spread using a sterile Drigalski spatula and incubated at a room temperature of 25 °C for 5 days. The resulting colonies were counted, and the total yeast count was calculated as log_10_ CFU/g [48].

Morphologically distinct colonies, based on color and shape, were subsequently transferred onto Blood agar and cultured under the same conditions. Pure cultures obtained from the isolates were subjected to microscopic examination and identified at the species level using a MALDI–TOF system (AUTOF MS 1000).

### 2.5. Statistical Analysis

Statistical analyses and comparisons of yeast groups based on specific parameters were performed by applying the Mann–Whitney U test for independent samples and the *t*-test. Correlations between yeast count and the physicochemical parameters of the cheeses (salt content, acidity, fat content, moisture, and ripening stage) were evaluated using Spearman’s and Pearson’s correlation coefficients, depending on the normality of the data distribution. Statistical significance was determined at a significance level of α = 0.05. Values of *p* ≤ 0.05 were considered statistically significant. Statistically significant differences and correlations between the studied parameters were visualized using charts in Microsoft Excel.

## 3. Results

In this study, the species composition of yeasts was analyzed in 50 industrial white brine cheese samples and 50 artisanal white brine cheese samples (Table S1 in Supplementary Materials). The yeast species were identified using a MALDI–TOF system, which confirmed the presence of the following species: *T. delbrueckii*, *D. hansenii*, *S.cerevisiae*, *C. sphaerica*, *C. zeylanoides*, *C. valida*, *C. lambica*, *G. candidum*, and *Rhodotorula* spp.

**Figure 1:**
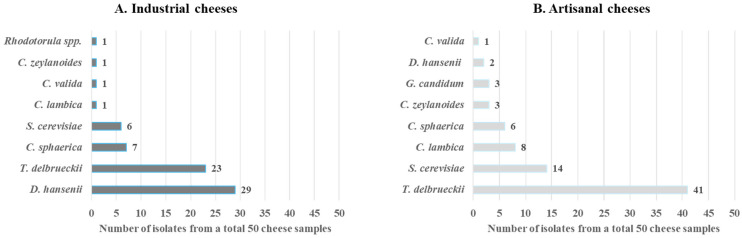
Species composition and yeast count isolated from (**A**) industrial white brined cheeses and (**B**) artisanal white brined cheeses.

The identified yeast species and their distribution are presented in Figure 1.

The results presented in Figure 1 indicate that the yeast species composition in industrial and artisanal cheeses is similar; however, there are significant differences in the predominant yeast species between the two examined categories of white brined cheeses. This indicates that certain yeast species are more predominant in artisanal cheeses, whereas others are more frequently associated with industrial cheeses.

In industrial cheeses, *D. hansenii* was the most frequently isolated species (58%), followed by *T. delbrueckii* (46%), with *Candida* spp. and *S. cerevisiae* detected at lower proportions. In contrast, in artisanal white brined cheeses, *T. delbrueckii* was isolated from 82% of the analyzed samples, followed by *S. cerevisiae* and *Candida* spp., while the proportion of *D. hansenii* in this category was significantly lower (4%).

The greatest deviation from the expected values was observed in *D. hansenii*, with statistically significant differences in its isolation frequency between the two cheese categories (*p* < 0.001). Statistically significant results were also observed in the isolation frequency of *S. cerevisiae* in both artisanal and industrial cheeses (*p* = 0.0365). In contrast, no statistically significant differences were found for *T. delbrueckii* (*p* = 0.105) and *Candida* spp.

Regarding the average yeast count (log_10_ CFU/g) in industrial and artisanal cheeses, *D. hansenii* and *T. delbrueckii* showed no significant differences (*p* = 0.101). The yeast count of *D. hansenii* ranged from 5.82 to 6.33 log_10_ CFU/g in artisanal and industrial products, respectively. Similarly, *T. delbrueckii* demonstrated an average yeast count of 5.00 log_10_ CFU/g in artisanal cheese samples and 5.07 log_10_ CFU/g in industrial cheeses.

**Figure 2:**
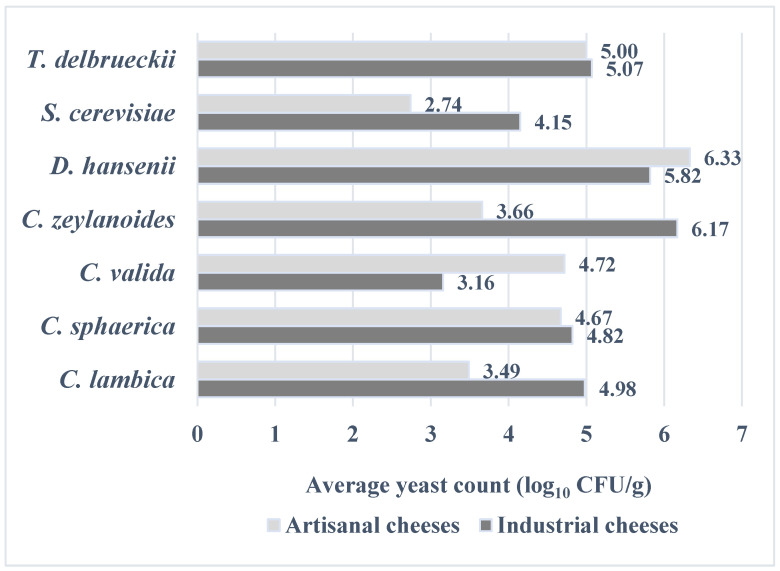
Yeast count (log_10_ CFU/g) in industrial and artisanal white brined cheese samples.

Significant differences were observed for *S. cerevisiae*, with the average yeast count showing higher values in industrial cheeses (4.15 log_10_ CFU/g) compared to artisanal cheeses (2.74 log_10_ CFU/g) (*p* = 0.0031). For *Candida* species, the yeast counts were similar, with the exception of *C. zeylanoides*. However, the limited number of isolates restricted the ability to draw well-defined conclusions about *Candida* spp. (Figure 2).

The results present the average yeast count of the isolated yeasts from a total of 100 cheese samples.

After identifying differences in the predominant species composition between industrial and artisanal cheeses, as well as variations in the yeast count, a correlation analysis was performed to detect relationships between the obtained results and the physicochemical characteristics of the analyzed samples. The studied physicochemical parameters of the white brine cheese samples, which could influence the species composition and yeast count, include salt and moisture content, acidity, fat content, and degree of maturity.

Firstly, the statistical significance of the differences in each of these parameters between the artisanal cheese samples and the industrially produced samples was analyzed. Applying the Mann–Whitney U test and the *t*-test, we found that there were no significant differences between the two categories of white brine cheeses with respect to the parameters of salt content, acidity, fat content, and maturity (*p* > 0.05). A statistically significant difference was observed in moisture content (*p* = 0.048), indicating that the production method of the sample set significantly affects only this physicochemical parameter. The correlation between moisture content in the samples and each of the other cheese characteristics was analyzed, revealing a relationship with the ripening stage (r = −0.30). More mature cheeses exhibited lower moisture content, which is expected, as moisture decreases during ripening. The other parameters (salt content, acidity, and fat content) did not show a significant correlation with moisture content.

After analyzing the influence of each characteristic individually on the detection of yeast species in the samples, a statistically significant correlation with the salt content of the cheese was identified. Salt content emerged as a key factor determining the predominant yeast species in both artisanal and industrial cheeses (Table 1). The acidity of the samples also stood out as a factor influencing the formation of the yeast species composition in white brined cheeses, while the moisture content showed a statistically significant correlation with the yeast species composition in industrial cheeses.

When analyzing the correlations between the physicochemical characteristics of the cheeses and the yeast count, a statistically significant relationship was observed only with the salt content. This trend was particularly pronounced in industrial cheeses, while none of the other characteristics exhibited a statistically significant relationship with the yeast count.

**Figure 3:**
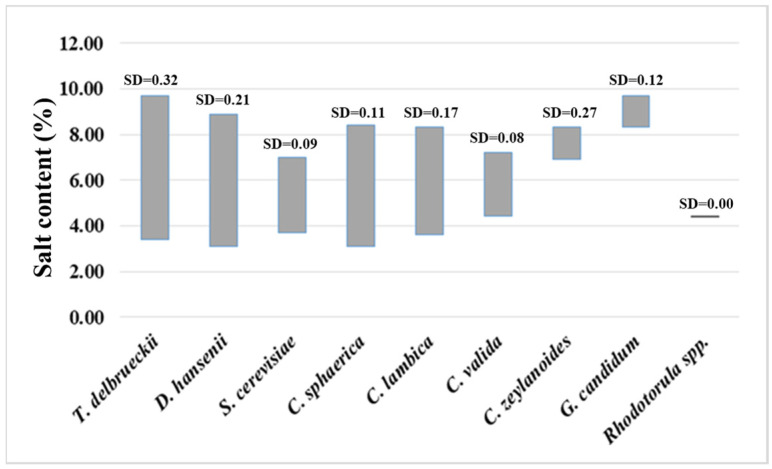
The salt tolerance of yeast species isolated from white brined cheese samples. SD—standard deviation.

**Figure 4:**
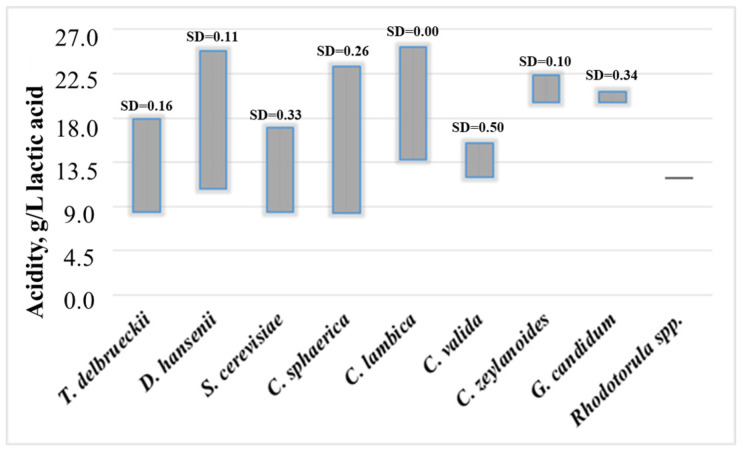
The acidity tolerance of yeast species isolated from white brined cheese samples SD—standard deviation.

**Figure 5:**
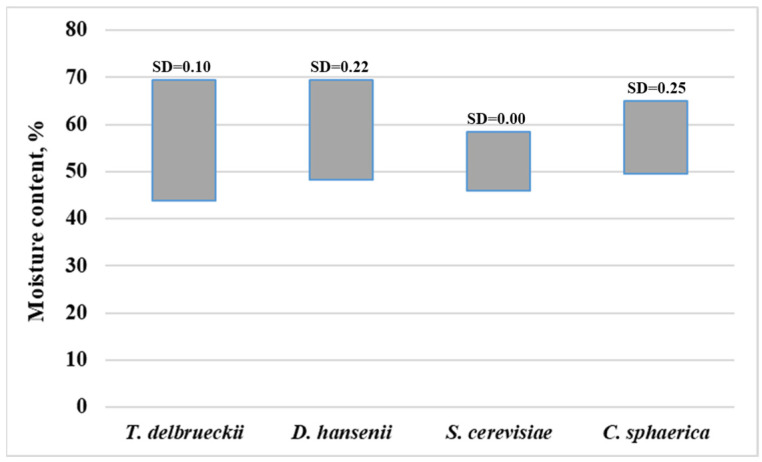
The influence of moisture content in industrial white brined cheeses on the formation of the yeast species composition. SD—standard deviation.

In relation to the results presented in Table 2, the salt and acidity tolerance range of yeast species isolated from cheese samples was investigated (Figure 3 and Figure 4), as well as a more in-depth analysis of the influence of moisture content in industrial cheeses on the formation of the yeast species composition (Figure 5).

The distribution of yeast species across different salt concentrations in cheese samples demonstrates statistically significant variability (Table 2, Figure 4). The yeast species *T. delbrueckii*, *D. hansenii*, *C. sphaerica*, and *C. lambica* exhibited a broader range of salt tolerance, growing in samples with salt concentrations ranging from 4% to 10%, indicating high adaptability. In contrast, *S. cerevisiae*, *C. valida*, and *C. zeylanoides* showed more consistent growth within a narrower range of 5% to 7%, suggesting specific physiological adaptation to moderate salt stress. For *G. candidum* and *Rhodotorula* spp., the limited number of isolates constrains the ability to identify clear trends. Notably, at the highest salt concentrations (≥9%), a predominance of *T. delbrueckii*, *D. hansenii*, and *G. candidum* was observed, while *S. cerevisiae* and *C. valida* did not grow under such conditions.

The acidity of white brined cheese also emerged as a critical factor influencing the species distribution of yeasts. Figure 4 reflects the trends in the tolerance of the isolated yeast species to the acidity of the cheese.

The results indicate that species such as *T. delbrueckii*, *D. hansenii*, *C. sphaerica*, and *C. lambica* are associated with a wider acidity range, suggesting their high adaptability. These species also represent the main isolates from white brined cheeses with high acidity. In the cheese samples, *T. delbrueckii* exhibited the widest tolerance range of acidity levels (8.40–25.20 g/L lactic acid), suggesting its adaptability to varying pH conditions. *S. cerevisiae*, typically associated with fermentative environments, displayed acidity tolerance within a narrower range (8.40–18.05 g/L lactic acid) and development at lower acidity levels. A similar trend was observed for the species *C. valida*.

The influence of moisture content in the cheese samples was examined with regard to the yeast species *T. delbrueckii*, *D. hansenii*, *S. cerevisiae*, and *C. sphaerica*. The remaining species were identified in only a single isolated result from the industrial cheeses analyzed in the study. Examining the relationship between this physicochemical parameter of the cheeses and the yeast species composition confirmed the previously noted trend. Specifically, the higher adaptability of *T. delbrueckii* and *D. hansenii* to a wide range of environmental factors—salt, acidity, and in this case, moisture content—was reaffirmed. At the same time, the narrower tolerance spectrum of *S. cerevisiae* and *C. sphaerica* to specific moisture levels in the cheeses was observed, with these species predominantly thriving at lower and moderate moisture levels, respectively.

**Figure 6:**
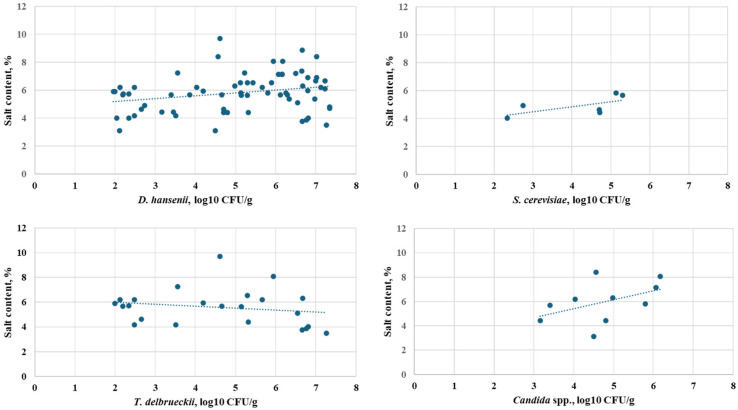
Scatter plot of the correlation between salt content (%) in industrial cheese samples and yeast count (log_10_ CFU/g).

Regarding the factors influencing the microbial count of yeasts in white brined cheeses, none of the examined physicochemical characteristics showed a clear dependence. The salt content in industrial cheeses was identified as a statistically significant correlating factor related to the formation of yeast counts (Figure 6). The scatter plots in Figure 6 present a correlation between salt content (%) and yeast count (log_10_ CFU/g) in industrial cheese samples, indicating that higher salt content is linked with specific yeast growth patterns. Overall, a positive trend is observed, suggesting that higher salt levels are generally associated with increased yeast growth. However, the strength of the correlation varies across subgroups. In some cases (e.g., top right and bottom right panels), the relationship is stronger, indicating that salt may promote yeast development under specific processing conditions. In others (e.g., bottom left), the effect is weak, suggesting additional factors may also influence yeast proliferation.

To investigate the relationship between the isolation frequency of specific yeast species (*T. delbrueckii*, *D. hansenii*, *S. cerevisiae*, *Candida* spp.) and the physicochemical characteristics of cheese (salt content, acidity, moisture, maturity, and fat content), a correlation analysis was conducted. The Pearson correlation coefficient (r) was used to determine the linear relationships between the number of isolates and the physicochemical characteristics.

Applying this analytical approach, in addition to salt content, acidity, and moisture content in the cheeses, the fat content was also identified as a characteristic influencing the development of certain yeast species. Fat content and salt concentration in cheeses influence the presence of *T. delbrueckii* in white brined cheeses. *T. delbrueckii* demonstrated a negative correlation with both salt content (−0.1687) and fat content (−0.2410), indicating that this yeast species is less frequently found in saltier and fattier cheeses.

The number of *D. hansenii* isolates showed correlations with the same physicochemical characteristics of the cheeses, but in contrast to *T. delbrueckii*, these correlations were positive with salt content (0.2239) and fat content (0.2714). This suggests that *D. hansenii* is more commonly found in saltier and fattier cheeses. A positive correlation was also observed between *D. hansenii* and acidity, which aligns with previous findings indicating the high tolerance of this species to acidity and salt content in white brined cheeses.

The results from Figure 3 and Figure 4 demonstrated that both *T. delbrueckii* and *D. hansenii* are highly adaptable yeast species that are isolated from cheeses with a broad range of salt content and acidity levels. At the same time, more detailed data on the distribution of yeast species in the cheeses highlighted a clear pattern: namely, *T. delbrueckii* tends to prevail and develop more frequently in cheeses with lower acidity and lower salt and fat content. In contrast, *D. hansenii* exhibits the opposite trend, being more frequently isolated from cheeses with high acidity and high salt and fat content.

For *S. cerevisiae*, negative correlations were recorded with salt content (−0.1484) and acidity (−0.1844), indicating that this species is more frequently isolated from cheeses with lower salt content and acidity. Regarding *Candida* spp., results showed a positive correlation with salt content (0.1078), suggesting a preference for saltier cheeses, and a slight negative correlation with acidity (−0.0724).

## 4. Discussion

Industrial white brined cheeses are typically produced under strictly controlled conditions, ensuring consistent product quality and microbial composition. In contrast, artisanal white brined cheeses are produced using traditional methods, often relying on natural fermentation and local microbial populations. Variability in raw milk composition and environmental conditions contributes to greater diversity in texture, flavor, and microbial profiles. The extent to which these technological differences influence yeast populations in brined cheeses remains a poorly studied area. Nevertheless, the main yeast species found in white brined cheeses have been well characterized in several recent studies [4,22,27,30,32,49,50], and the results obtained in our study confirmed this microbial profile. The presence of yeast species from the genera *Debaryomyces*, *Geotrichum*, *Kluyveromyces*, *Pichia*, *Rhodotorula*, *Saccharomyces*, *Torulaspora*, *Candida*, and *Yarrowia* in white brined cheeses has been frequently reported.

The main yeast species identified in our study—*D. hansenii*, *T. delbrueckii*, and *Candida* spp.—have been widely reported in the literature with similar patterns. *D. hansenii* is known for its high salt tolerance and predominance in industrial cheeses [13,50,51,52], while *T. delbrueckii* tends to be more frequent in artisanal products, reflecting differences in production methods [20]. Although *Candida* spp. are often considered contaminants, recent studies suggest their possible role in early ripening stages and flavor development [13,35]. These observations align with previous research showing that production technology, salt content, and ripening conditions strongly influence yeast community composition in white brined cheeses.

Analysis of 50 industrial and 50 artisanal cheese samples revealed differences in yeast species composition, with a significant predominance of *D. hansenii* in industrial cheeses and sporadic isolation in artisanal samples. The greatest deviation from the expected values was observed in *D. hansenii*, which may suggest that this species is either favored or inhibited depending on the production technology of white brined cheeses. This is likely due to its resistance to high salt content and its role in industrial production.

The species *T. delbrueckii* and *S. cerevisiae* were isolated from both types of samples, but with a twofold higher frequency in farmhouse cheeses. No significant differences were observed for *Candida* spp. These findings indicate that the overall yeast species composition in the two cheese categories is similar; however, significant differences are evident in the predominant species. The main differences in the yeast species composition are primarily explained by variations in raw materials, the use of pasteurization, curdling temperature (30–38 °C), and physicochemical parameters related to salt concentration (7–16% NaCl), ripening, and acidity [51]. Salt plays a significant role in shaping the microbial dynamics within cheese [13]. It influences microbiological processes by exerting a selective effect on different microbial species. However, salt itself is often a source of microbial contamination. Therefore, thermal treatment (e.g., dry heating) of the salt prior to use is recommended to reduce the risk of introducing undesirable microorganisms. The storage period and hygiene standards during cheese production also influence the microbial profile of cheeses.

Significant differences in the predominant yeast species have also been reported in an earlier study, which examined yeast isolates in white brined cheese from three different dairies in Denmark. The results showed that in one of the dairies, *T. delbrueckii* was the most frequently identified isolate, while *D. hansenii* was detected sporadically. In contrast, in another dairy, *D. hansenii* was the predominant isolate. The authors concluded that yeast species composition in cheeses is influenced both by technological aspects of the production process and the resulting physicochemical characteristics of the final product [52].

Our analysis showed no significant differences in the physicochemical parameters of farmhouse and industrial cheeses, with most values falling within similar ranges in both groups. Differences in moisture content emerged as the only statistically significant characteristic distinguishing farmhouse from industrial cheeses. It is likely that the yeast species composition in cheeses is more strongly influenced by technological differences in the production process, particularly milk pasteurization. Many authors identify pasteurization as a key factor influencing yeast composition and count in cheese [2]. Previous studies have reported that the only statistically significant difference between cheeses made from raw milk and pasteurized milk is the higher moisture content in artisanal cheeses [53,54]. This may be attributed to the effect of heat treatment on the capacity of the cheese protein to hold water [55]. Higher moisture content can lead to the development of off-flavors due to the formation of soluble breakdown products of acids, sugars, proteins, and lipids [56,57].

In industrial production, milk is pasteurized before cheese production at 75 °C for 15 s, effectively controlling microbiological food safety risks. Besides pathogen inactivation, this heat treatment is expected to eliminate viable vegetative cells in the processed milk. Some studies have reported that the yeasts isolated from white brined cheese produced with pasteurized milk primarily originate from environmental contamination during production stages following heat treatment. Production facilities, especially contaminated air, have been identified as major sources of yeast in white brined cheeses. The highest yeast concentrations were detected on work surfaces in the milk reception area, the cheese production zone, and in air samples. A recent study identified the following yeasts from air and surface samples in commercial and traditional Polish dairy processing facilities: *D. hansenii*, *G. candidum*, *Rhodotorula* spp., *Yarrowia lipolytica*, and *Candida* spp. Additionally, rennet can be a potential source of microbial contamination if it is not obtained through sterile filtration [58].

In the production of artisanal cheeses, raw milk is traditionally used, incorporating natural whey cultures as starters [2,59]. Studies on artisanal cheeses suggest that species such as *T. delbrueckii*, *C. catenulata*, and *D. hansenii* are likely important components of the cheese’s natural flora [59]. Yeasts in artisanal cheeses primarily originate from raw milk, which naturally contains microorganisms from the animals’ skin, their feed, and the milking equipment. The air and environment in the dairy, including wooden shelves and equipment, also contribute to the unique microbial profile of artisanal cheeses [8,60]. During the ripening process, yeasts develop on the cheese rind, with some cheesemakers intentionally relying on the natural flora [38]. The hands of the cheesemakers and the addition of specific starter cultures also contribute to the unique taste and aroma of the cheese. All these factors collectively shape the distinctive character of artisanal cheeses.

Several studies have shown that yeasts such as *D. hansenii*, *K. lactis*, and *Y. lipolytica* positively contribute to flavor development during the ripening stage [4,59]. Moreover, in recent years, the innovative characteristics of yeasts, such as their probiotic potential, functional properties, and the production of bioactive compounds, have gained increasing attention [60,61,62,63,64,65]. In this regard, *T. delbrueckii* has attracted significant scientific interest [20,66,67,68]. The use of probiotic yeasts can make the product a candidate for functional food. Many recent studies on yeasts isolated from cheeses have shown that these microorganisms are promising probiotics [68] and demonstrated that various types of yeasts in cheeses significantly extend the product’s shelf life and protect it from contamination with *Salmonella enterica* [69]. In addition to their technological and probiotic roles, the prevalence of different yeast species may also influence the sensory characteristics of cheese. *D. hansenii*, more frequently isolated from industrial cheeses, is associated with the formation of savory and salty flavor notes [13], whereas *T. delbrueckii* and *S. cerevisiae*, dominant in artisanal cheeses, contribute to more complex and fruity aromatic profiles [20,66].

Interestingly, in industrial cheese production, yeasts are considered microbial contaminants, even though they are sometimes part of the secondary microbiota [22]. It is believed that their metabolic activity can lead to changes in organoleptic properties, reduced shelf life, and deteriorated quality of dairy products [70]. The negative impact of yeasts on white brined cheese is primarily associated with the development of bitterness, resulting from the proteolytic degradation of cheese proteins. Additionally, yeasts can induce undesirable changes in the brine, most notably the formation of a surface film known as Mycoderma [71]. The likely reasons why yeasts might lead to undesirable characteristics in white brined cheeses are their excessive proliferation, which can result in the production of certain undesirable metabolic compounds. Other authors also suggest that the adverse effects in dairy products are not due to the presence of specific yeast species, but rather to the stimulation of their excessive proliferation [13].

Regarding yeast counts, we recorded similar average counts for *D. hansenii* in industrial and artisanal cheeses (5.82 log_10_ CFU/g and 6.33 log_10_ CFU/g, respectively). For *T. delbrueckii*, the values were also comparable—5.07 log_10_ CFU/g in industrial samples and 5.00 log_10_ CFU/g in artisanal cheeses. For *Candida* spp., the average counts ranged between 3.16 log_10_ CFU/g and 6.17 log_10_ CFU/g, with no statistically significant differences. The results from other studies investigating yeast communities in white brined cheeses report similar values, with the yeast species count typically ranging between 2 log_10_ CFU/g and 6 log_10_ CFU/g [22,30,72].

The yeast count of *S. cerevisiae* varied in both categories of white brined cheeses (2.74–4.14 log_10_ CFU/g). A significantly higher yeast count of *S. cerevisiae* was observed in industrial cheeses compared to artisanal ones, with statistically significant differences. The industrial cheese samples analyzed in our study did not include starter cultures containing *S. cerevisiae*, which may explain the observed differences. The presence of this yeast species is likely due to spontaneous fermentation or cross-contamination during the production process. Incomplete pasteurization or post-processing contamination may also contribute to the presence of *S. cerevisiae* in industrial cheeses. Some studies suggest that even after thermal treatment, environmental factors such as contaminated equipment and air can introduce yeasts into the product [22,59]. Additionally, the reduction of microbial diversity through pasteurization may facilitate the overgrowth of *S. cerevisiae* when reintroduced, as competition from other microorganisms is diminished [13,53]. The higher yeast count of *S. cerevisiae* in industrial cheeses compared to farm-produced ones can be attributed to several factors. Industrial production conditions, such as controlled temperature, humidity, and pH, create an optimal environment for yeast development when it is introduced secondarily into the product. Additionally, milk pasteurization in industrial processes reduces competition from other microorganisms, further stimulating the growth of *S. cerevisiae*. In contrast, the use of raw milk in artisanal cheeses maintains a more diverse microbial community, which restricts the growth of *S. cerevisiae* through competitive exclusion.

The applied statistical analyses showed that the factors most strongly influencing the presence of certain yeast species in cheeses are salt content, acidity, and moisture. Salt content was a statistically significant factor for the yeast count in industrial cheese samples. Other studies also report that the presence of certain yeast species in cheeses is highly dependent on physicochemical characteristics such as salt content and acidity [13].

Analyzing the influence of physicochemical factors on the development of specific yeast species revealed some significant differences. The species *D. hansenii* was more commonly found in saltier and fattier cheeses, while at the same time showing tolerance to a wide range of acidity and salt content in the cheese samples. A positive correlation was also observed between *D. hansenii* and acidity, which aligns with previous findings. Fröhlich-Wyder (2019) reports that *D. hansenii* exhibits high halotolerance and can survive up to 20–24% (*w*/*w*) NaCl, along with the ability to grow at low pH and utilize lactate as a primary carbon source [13]. This species can be found on the surface of a wide range of cheeses and in the brines used for their salting, with the brining stage being the most likely point of introduction of *D. hansenii* into cheeses [4].

In the industrial and artisanal cheese samples we studied, *T. delbrueckii* was ubiquitously isolated from both categories. No statistically significant difference was observed in the presence of *T. delbrueckii* between industrial and artisanal cheeses (*p* > 0.05), suggesting that the production technology does not have a major influence on the distribution of this yeast species. *T. delbrueckii* exhibited a wide range of tolerance to salt concentration and acidity in the cheeses. At the same time, this species demonstrated a negative correlation with both salt content and fat content, indicating that this yeast species is less frequently found in saltier and fattier cheeses. The ability of yeasts to survive in a wide range of chemical conditions, even those similar to the gastrointestinal tract, has been widely observed in strains of *Candida*, *Debaryomyces*, and *Torulaspora*, showing results comparable to both one another and the control strain [73]. In this regard, Zivkovic (2015) describes *T. delbrueckii* as the most resistant strain under conditions simulating gastric juice, highlighting the low survival rate of *S. cerevisiae* [73,74]. Results from a study by Andrade (2021) show that *T. delbrueckii* is an exceptionally interesting yeast in the context of probiotics, positively influencing the composition of desirable volatile compounds in cheeses [20].

Similar to the results obtained by [75], *S. cerevisiae* was more frequently isolated from cheeses with lower salt content and acidity. Overall, a more in-depth analysis of the factors that had a statistically significant influence on yeast species composition (moisture, acidity, and salt content) showed that *S. cerevisiae* demonstrated the narrowest range of tolerance to the examined factors, as the species was predominantly identified at intermediate values of the studied parameters. González-Hernández (2005) confirms that *S. cerevisiae* has significantly lower salt tolerance compared to *D. hansenii* [75].

Regarding *Candida* spp., a recent study reported the presence of the genus *Candida* in higher abundance and frequency than previously documented. A large heterogeneity of *Candida* species was detected in cheeses, and while they are currently considered contaminants [35], some species may still play an unexplored role in the early stages of cheese ripening [13]. *C. sphaerica* and *G. candidum* were more frequently detected in farmhouse products than expected, which may be attributed to the natural ripening process and the absence of controlled starter cultures.

These observations suggest that salt content and acidity may play a crucial role in shaping the yeast community composition in cheese. The results highlight the significant influence of salt concentration on the distribution of yeast species in cheese. The broad salt tolerance observed in *T. delbrueckii* and *D. hansenii* suggests that these species are well-adapted to fluctuating environmental conditions, making them resilient across different cheese production systems. The ability of *T. delbrueckii* to persist under different environmental conditions suggests its potential role in various types of cheese, while the dominance of *D. hansenii* in industrial cheese samples highlights its importance in industrial production as a secondary contaminant in cheeses. The strong presence of *D. hansenii* in samples with high salt content is consistent with previous studies, which classify it as a halotolerant yeast species commonly found in salty cheeses. Meanwhile, the limited salt tolerance of *S. cerevisiae* suggests that its presence may be restricted to cheeses with lower salt levels, potentially influencing the dynamics of fermentation.

## 5. Conclusions

Understanding the entire cheese microbiome is essential to gaining insight into how the flavor and other sensory characteristics of each cheese variety develop and to maintaining control over the overall quality of the cheese. In conclusion, this study provides a comprehensive analysis of the yeast species composition and yeast count in industrial and artisanal white brined cheeses, highlighting differences influenced by production technology and environmental factors. The results confirmed that while the overall yeast community structure in both cheese types is similar, significant differences were observed in the predominance of specific yeast species. *D. hansenii* was more frequently isolated from industrial cheeses, whereas *T. delbrueckii* and *S. cerevisiae* were more prevalent in artisanal cheeses, reflecting the impact of raw milk use and natural fermentation processes.

The findings suggest that salt content, acidity, and moisture are the primary physicochemical factors shaping the yeast community in white brined cheeses. *D. hansenii* demonstrated high halotolerance and adaptability to varying acidity levels, explaining its dominance in high-salt environments. In contrast, *S. cerevisiae* exhibited a narrower tolerance range, with higher counts in industrial cheeses likely resulting from controlled production conditions and reduced microbial competition due to pasteurization.

These insights enhance our understanding of the microbial ecology of white brined cheeses and provide a basis for optimizing production processes to improve product quality and consistency. Further research focusing on the functional role of yeasts, particularly their contribution to flavor development and potential probiotic properties, could open new opportunities for developing high-value dairy products. These findings have practical implications for cheese producers aiming to optimize microbial communities to enhance flavor, texture, and overall product quality, bridging scientific understanding with industry applications.

## Figures and Tables

**Table 1:** Correlations between the physicochemical characteristics of artisanal and industrial cheeses and their influence on the species composition and yeast count in the samples, assessed using statistical methods.

	Correlation Analysis of Differences in Physicochemical Parameters Between Artisanal and Industrial Cheeses (Mann–Whitney U Test/*t*-Test)				
Total cheese samples	salt content *p* = 0.700	acidity *p* = 0.381	fat content *p* = 0.101	moisture content *p* = 0.048	degree of maturity *p* = 0.155
	Correlation between physicochemical parameters and the species composition of isolated yeasts (ANOVA test)				
Total cheese samples	* salt content *p* = 0.0074	* acidity *p* = 0.0028	fat content *p* = 0.351	moisture content *p* = 0.585	degree of maturity *p* = 0.634
Industrial cheese samples	* salt content *p* = 0.016	acidity *p* = 0.721	fat content *p* = 0.296	* moisture content *p* = 0.0057	degree of maturity *p* = 0.647
Artisanal cheese samples	* salt content *p* = 0.022	* acidity *p* = 0.0055	fat content *p* = 0.748	moisture content *p* = 0.0788	degree of maturity *p* = 0.320
	Correlation between physicochemical parameters and yeast count (log_10_ CFU/g) (Spearman’s rank correlation coefficient)				
Total cheese samples	salt content *p* = 0.414	acidity *p* = 0.427	fat content *p* = 0.561	moisture content *p* = 0.910	degree of maturity *p* = 0.086
Industrial cheese samples	* salt content *p* = 0.029	acidity *p* = 0.52	fat content *p* = 0.71	moisture content *p* = 0.51	degree of maturity *p* = 0.78
Artisanal cheese samples	salt content *p* = 0.91	acidity *p* = 0.81	fat content *p* = 0.727	moisture content *p* = 0.80	degree of maturity *p* = 0.909

* Parameters for which statistical significance was observed. *p*-values ≤ 0.05 were considered statistically significant.

**Table 2:** Correlation by number of isolates regarding a specific yeast species and the physicochemical characteristics of the total cheese samples.

Yeast Species	Salt Content	Acidity	Fat Content
*T. delbrueckii*	r = *−0.1687*	-	**r = −*0.2410***
*D. hansenii*	***r = 0.2239***	r = *0.1761*	**r = *0.2714***
*S. cerevisiae*	r = *−0.1484*	r = *−0.1844*	-
*Candida* spp.	r = *0.1078*	r = *−0.0724*	-

## Data Availability

The original contributions presented in this study are included in the article/Supplementary Materials. Further inquiries can be directed to the corresponding author.

## Supplementary Materials

The following supporting information can be downloaded at: https://www.mdpi.com/article/10.3390/microorganisms13091965/s1, Table S1: Analysis of 100 cheese sample (50 industrial cheeses and 50 artisanal cheeses)—results from isolated yeast species, yeast count and physicochemical properties.
