# Supplementary material for: Yeast Ecology in White Brined Cheeses: Correlations with Physicochemical Parameters in Artisanal and Industrial Products
Source: Microorganisms. 2025 Aug 22;13(9):1965. doi: 10.3390/microorganisms13091965 (PMC12471494; doi:10.3390/microorganisms13091965)
Supplement: Supplementary file 1 [file microorganisms-13-01965-s001.zip › microorganisms-3769655-supplementary.pdf]

Supplementary:

Table S1. Analysis of 100 cheese sample (50 industrial cheeses and 50 artisanal cheeses) - results from isolated yeast species, yeast count and physicochemical properties

**Sample number**	**Technological specification**	**Isolated yeasts species**	**Yeast count (Log10 CFU/g)**	**Salt content (%)**	**Acidity (ºT)**	**Moisture content (%)**	**Maturity (oШ)**	**Fat content (%)**
1	artisanal	*S. cerevisiae*	2.17±0.18	4.20±0.20	152.90±0.65	54.60±2.12	30	54.00
		T. delbrueckii	4.36±0.08					
2	artisanal	*S. cerevisiae*	2.12±0.54	3.69±0.02	176.70±0.70	56.01±1.10	50	54.60
		T. delbrueckii	8.31±0.36					
3	artisanal	*S. cerevisiae*	2.56±0.45	5.73±0.32	187.00±4.34	41.64±0.54	90	38.60
		T. delbrueckii	9.93±0.11					
4	artisanal	*T. delbrueckii*	7.12±0.08	5.49±0.22	156.07±3.40	57.50±1.69	55	50.60
5	artisanal	*T. delbrueckii*	5.84±0.30	5.85±0.15	183.33±0.65	56.87±0.39	50	30.10
6	artisanal	*T. delbrueckii*	2.66±0.25	2.37±0.05	162.00±0.50	42.71±2.43	100	34.00
7	artisanal	*T. delbrueckii*	3.00±0.40	5.53±0.34	189.33±3.50	46.85±3.19	90	31.00
		C. lambica	2.64±0.02					
8	artisanal	*T. delbrueckii*	5.84±0.06	3.39±0.50	166.70±4.34	49.95±0.11	85	35.00
9	artisanal	*T. delbrueckii*	3.94±0.34	3.86±0.04	114.70±3.20	37.06±1.32	70	26.20
		C. lambica	2.16±0.26					
10	artisanal	*T. delbrueckii*	5.61±0.12	3.62±0.40	152.70±0.30	59.13±1.06	75	53.80
		C. lambica	3.20±0.09					
11	artisanal	*T. delbrueckii*	2.70±0.20	3.55±0.63	100.00±4.45	51.08±2.35	50	43.90
12	industrial	*T. delbrueckii*	7.26±0.19	3.51±0.85	140.00±0.50	58.75±1.87	85	31.50

13	industrial	*T. delbrueckii*	2.48±0.50	6.20±0.00	160.00±0.20	57.09±2.67	45	32.60
14	artisanal	*C. sphaerica*	6.03±0.05	6.08±0.40	137.33±2.20	65.20±2.15	100	51.70
15	industrial	*S. cerevisiae*	2.74±0.18	4.91±0.45	161.33±0.60	58.51±2.03	50	47.00
16	artisanal	*C. sphaerica*	5.75±0.98	8.30±0.20	177.40±5.05	51.14±0.85	60	40.90
17	artisanal	*S. cerevisiae*	2.19±1.04	7.72±0.06	165.40±3.60	55.54±0.93	90	21.40
		T. delbrueckii	4.54±0.22					
18	artisanal	*T. delbrueckii*	3.19±0.40	7.25±0.00	199.40±0.55	56.36±0.29	105	43.50
19	artisanal	*T. delbrueckii*	6.13±0.06	4.91±0.35	116.70±0.45	50.81±2.21	95	28.50
20	artisanal	*T. delbrueckii*	5.18±0.09	4.36±0.10	170.60±2.35	51.75±2.73	60	44.60
21	artisanal	*T. delbrueckii*	5.11±0.55	3.51±0.25	129.40±2.20	58.67±3.34	55	29.00
22	artisanal	*S. cerevisiae*	2.32±0.20	3.51±0.80	93.33±5.30	54.73±1.01	40	51.90
		T. delbrueckii	2.64±0.20					
23	artisanal	*S. cerevisiae*	2.89±0.40	6.47±0.15	108.70±0.40	59.40±1.15	30	56.70
		C. sphaerica	4.73±0.09					
24	artisanal	*S. cerevisiae*	2.95±0.60	3.62±0.08	164.70±0.00	50.15±0.57	60	40.10
		C. sphaerica	5.28±0.08					
25	artisanal	*T. delbrueckii*	3.87±0.03	4.29±0.70	114.60±4.40	51.55±2.23	70	44.40
26	industrial	*T. delbrueckii*	4.19±0.06	5.93±0.65	142.00±2.25	58.69±4.42	65	44.80
27	artisanal	*T. delbrueckii*	5.20±0.60	4.99±0.23	166.70±2.80	51.01±0.40	90	55.10
28	artisanal	*T. delbrueckii*	5.72±0.19	7.10±0.67	134.00±4.70	52.77±2.74	90	47.60

29	industrial	*T. delbrueckii*	2.48±0.19	4.17±0.38	168.70±0.50	52.15±1.56	50	51.20
30	industrial	*T. delbrueckii*	3.51±0.02	4.17±0.20	140.00±0.05	51.01±0.88	50	52.20
31	artisanal	*S. cerevisiae*	2.98±0.50	6.70±0.64	167.33±0.80	46.15±5.35	100	53.80
		T. delbrueckii	5.17±1.00					
32	artisanal	*S. cerevisiae*	3.63±0.78	3.78±0.06	115.40±3.30	50.03±7.40	110	50.00
33	artisanal	*T. delbrueckii*	5.27±0.06	6.00±0.50	154.00±2.70	43.36±2.18	95	63.60
34	artisanal	*T. delbrueckii*	4.98±0.54	4.99±0.22	174.00±3.44	50.44±3.40	115	48.40
35	artisanal	-	-	3.24±0.11	132.60±3.00	54.45±1.54	60	48.30
36	artisanal	*C. valida*	4.72±0.55	7.21±0.04	172.00±0.60	53.65±1.43	55	53.90
		T. delbrueckii	4.13±0.02					
37	industrial	*C. valida*	3.16±0.08	4.44±0.40	132.62±0.08	50.14±3.49	40	57.20
		Rhodotorula spp.	3.45±0.38					
38	industrial	*S. cerevisiae*	5.13±0.03	5.81±0.86	187.40±4.40	55.47±0.67	25	25.80
		C. sphaerica	5.80±0.36					
39	industrial	*T. delbrueckii*	5.32±0.09	4.41±0.00	148.00±8.00	65.06±2.98	45	31.50
		S. cerevisiae	4.71±0.89					
		C. sphaerica	4.80±0.04					
40	industrial	*D. hansenii*	2.11±0.12	3.12±0.00	92.00±0.85	49.57±2.98	50	48.60
		C. sphaerica	4.50±0.34					
41	industrial	*S. cerevisiae*	4.70±0.40	4.64±0.40	156.00±0.00	46.01±3.49	215	31.50
		T. delbrueckii	2.65±0.77					

42	industrial	*S. cerevisiae*	2.34±0.23	4.01±0.45	194.00±2.90	56.06±0.61	95	60.30
		D. hansenii	2.04±0.02					
43	industrial	*T. delbrueckii*	6.66±0.51	3.76±0.35	152.00±3.50	49.99±0.73	65	50.00
44	artisanal	*T. delbrueckii*	5.34±0.19	3.97±0.60	232.60±3.80	48.07±2.11	70	37.60
		C. sphaerica	5.46±1.02					
45	industrial	*S. cerevisiae*	5.30±0.80	5.65±0.00	122.00±1.80	50.64±3.04	65	44.60
		T. delbrueckii	5.14±0.02					
46	artisanal	*S. cerevisiae*	2.89±0.05	6.47±0.07	108.70±0.40	59.40±2.21	30	56.70
		T. delbrueckii	4.73±0.20					
47	artisanal	*T. delbrueckii*	6.13±0.03	5.85±0.22	183.33±0.55	56.87±0.50	50	30.10
48	industrial	*T. delbrueckii*	6.54±0.09	5.11±0.20	144.00±0.70	43.76±2.93	65	54.20
49	industrial	*T. delbrueckii*	6.77±0.11	3.86±0.05	196.00±0.75	57.84±2.16	45	53.40
50	industrial	*T. delbrueckii*	6.81±0.35	4.01±0.40	162.00±0.00	58.35±1.65	90	51.60
51	industrial	*T. delbrueckii*	2.19±0.10	5.69±0.85	152.00±3.60	56.28±3.23	60	46.90
52	artisanal	*T. delbrueckii*	2.66±0.05	7.01±0.00	236.00±4.00	45.64±1.43	70	45.10
53	artisanal	*S. cerevisiae*	0.95±0.06	6.08±0.06	214.00±2.90	41.64±0.83	90	38.60
		T. delbrueckii	1.74±0.03					
54	artisanal	*S. cerevisiae*	5.12±0.34	5.96±0.50	200.50±0.00	59.13±1.17	75	53.80
		T. delbrueckii	3.40±0.55					

		C. lambica	3.16±0.45					
55	industrial	*T. delbrueckii*	5.29±0.67	6.55±0.75	188.00±3.00	61.30±0.71	70	62.50
56	industrial	*T. delbrueckii*	4.66±0.82	5.69±0.20	166.00±0.00	61.34±0.83	45	75.00
		D. hansenii	3.86±0.35					
57	industrial	*T. delbrueckii*	5.94±0.02	8.07±0.30	248.00±6.00	69.37±1.17	75	75.00
		C. zeylanoides	6.17±0.16					
58	industrial	*T. delbrueckii*	2.00±0.77	5.90±0.50	276.00±2.00	69.34±0.71	40	53.50
		D. hansenii	1.96±0.92					
59	industrial	*T. delbrueckii*	4.61±0.07	9.70±0.00	160.00±3.40	69.34±1.63	48	50.00
60	artisanal	*G. candidum*	4.46±0.03	8.31±0.55	218.00±4.00	55.25±1.17	45	35.80
		T. delbrueckii	5.12±0.20					
		C. lambica	3.12±0.23					
		C. zeylanoides	4.02±0.30					
61	artisanal	*G. candidum*	3.22±0.09	6.55±0.75	254.00±0.50	60.08±1.65	60	62.62
		T. delbrueckii	3.12±0.04					
		C. lambica	4.07±0.24					
62	artisanal	*T. delbrueckii*	3.07±0.05	6.90±0.25	230.00±0.60	54.72±0.04	65	61.84
		C. zeylanoides	3.64±0.26					
63	artisanal	*T. delbrueckii*	4.65±0.52	6.90±0.00	212.00±2.00	37.82±0.23	185	45.83
64	artisanal	*T. delbrueckii*	3.72±0.11	8.19±0.40	260.00±3.40	49.19±1.18	40	44.28

65	industrial	*T. delbrueckii*	5.67±0.09	6.20±0.14	214.00±5.00	50.19±2.48	40	21.08
66	artisanal	*T. delbrueckii*	4.77±0.56	6.67±0.16	198.00±0.40	53.65±2.14	75	62.00
		C. lambica	4.16±0.02					
67	artisanal	*T. delbrueckii*	2.90±0.06	7.14±0.50	222.00±2.00	60.81±1.21	55	53.90
		C. lambica	3.12±0.05					
		C. zeylanoides	3.33±0.08					
68	artisanal	*G. candidum*	4.12±0.11	7.37±0.04	230.00±0.60	50.34±1.05	165	45.30
		T. delbrueckii	4.11±0.24					
		C. lambica	3.45±0.03					
69	artisanal	*T. delbrueckii*	4.02±0.60	8.31±0.16	280.00±3.00	59.82±1.16	165	62.00
		C. lambica	5.12±0.70					
70	industrial	*D. hansenii*	2.20±0.08	5.73±0.40	216.00±1.50	59.76±0.26	75	75.00
		T. delbrueckii	2.34±0.02					
71	industrial	*T. delbrueckii*	6.67±0.30	6.32±0.25	260.00±1.50	56.93±2.23	35	40.63
		C. lambica	4.98±0.55					
72	industrial	*D. hansenii*	6.15±0.18	7.14±0.25	208.00±0.00	60.81±0.75	40	40.82
		C. sphaerica	6.07±0.05					
73	artisanal	*D. hansenii*	6.23±0.09	8.01±0.70	236.00±0.50	59.76±2.14	50	47.21
		C. sphaerica	3.12±0.14					
74	industrial	*D. hansenii*	6.16±0.19	6.20±0.56	258.00±1.50	50.58±1.21	70	30.35
		T. delbrueckii	2.12±0.05					
		C. sphaerica	4.03±1.02					
75	artisanal	*T. delbrueckii*	5.98±0.23	5.73±0.22	206.00±0.50	58.93±1.05	50	42.00
76	industrial	*D.hansenii*	5.90±0.55	6.55±0.00	166.00±0.00	69.34±1.16	80	65.23

77	industrial	*D. hansenii*	6.34±0.56	5.38±0.10	172.00±2.50	59.21±0.26	65	62.51
78	industrial	*D. hansenii*<br>*T. delbrueckii*	5.22±0.06<br>3.56±0.06	7.25±0.15	240.00±1.50	57.44±2.23	105	49.93
79	industrial	*D. hansenii*<br>*C. sphaerica*	6.29±0.08<br>3.40±0.25	5.69±0.65	196.00±1.50	52.230.75	75	46.05
80	industrial	*D. hansenii*	5.12±0.65	6.55±0.00	150.00±2.00	50.43±1.47	45	43.37
81	industrial	*D. hansenii*<br>*C. sphaerica*	7.02±0.11<br>4.56±0.29	8.42±0.44	200.00±0.00	54.73±0.33	105	43.07
82	industrial	*D. hansenii*<br>*C. sphaerica*	6.66±0.77<br>5.03±0.25	8.89±0.20	156.00±1.00	48.35±1.02	60	38.72
83	industrial	*D. hansenii*	7.02±0.90	6.90±0.60	136.00±0.00	48.29±0.66	70	46.41
84	industrial	*D. hansenii*	7.12±0.45	6.20±0.22	130.00±2.00	50.58±1.73	60	59.69
85	industrial	*D. hansenii*	6.97±0.33	5.38±0.10	228.00±2.20	57.89±0.66	60	43.93
86	industrial	*D. hansenii*	6.80±0.98	5.97±0.02	190.00±1.00	54.98±0.35	75	45.54
87	industrial	*D. hansenii*	7.23±0.45	6.67±0.55	240.00±0.00	51.88±2.86	75	48.83
88	industrial	*D. hansenii*	7.34±1.03	4.80±0.40	184.00±1.50	57.84±0.76	80	64.04
89	industrial	*D. hansenii*	6.65±0.55	7.37±0.19	138.00±1.50	57.08±1.23	55	93.20
90	industrial	*D. hansenii*	6.12±0.19	5.69±1.02	176.00±0.00	66.78±3.02	40	76.75
91	industrial	*D. hansenii*	5.43±0.34	6.55±0.30	242.00±0.45	56.17±1.57	110	46.77
92	artisanal	*T. delbrueckii*	6.13±0.06	4.91±0.65	116.70±0.50	50.81±0.38	95	28.50
93	artisanal	*D. hansenii*<br>*C. sphaerica*	6.43±0.05<br>3.12±0.04	8.01±0.44	236.00±2.50	59.76±2.28	50	50.00

94	artisanal	*S. cerevisiae*	3.03±0.09	5.17±0.20	167.33±0.48	46.15±1.15	100	50.00
		T. delbrueckii	5.70±1.10					
95	industrial	*S. cerevisiae*	3.70±0.30	3.90±0.25	118.20±1.00	53.70±0.55	100	50.00
96	industrial	*T. delbrueckii*	5.45±0.06	6.10±0.40	154.00±3.00	43.36±2.50	95	63.60
97	industrial	*S. cerevisiae*	3.06±0.05	4.01±0.82	120.45±0.55	55.20±3.12	105	50.00
98	industrial	*T. delbrueckii*	5.77±0.23	5.90±0.70	154.00±3.00	43.36±1.47	95	63.60
99	industrial	*D. hansenii*	7.23±0.70	6.10±0.35	135.00±2.20	52.58±0.90	65	59.20
100	industrial	*D. hansenii*	6.80±0.04	6.90±0.32	120.00±0.40	50.00±2.00	70	46.40
